# Is human enhancement intrinsically bad?

**DOI:** 10.1007/s11019-021-10003-w

**Published:** 2021-01-18

**Authors:** Karolina Kudlek

**Affiliations:** 1 Department of Philosophy, University of Twente, Enschede, The Netherlands; 2 Institute of Philosophy, Zagreb, Croatia

**Keywords:** Human enhancement, Intrinsic value, Intrinsic badness, Bioconservatives, Moral permissibility

## Abstract

A pertinent concern in the human enhancement debate is that human enhancement technologies (HET) are intrinsically bad and, hence, morally impermissible. This article evaluates the related claims about the intrinsic badness of HET by looking into philosophical theories of intrinsic value. It investigates how well-established conceptions of intrinsic value map onto typical bioconservative arguments about HET's intrinsic badness. Three predominant variants of these arguments are explored and found wanting: (i) HET are intrinsically bad owing to their unnaturalness; (ii) the pursuit of HET reveals intrinsically bad character (“the desire for mastery”); and (iii) HET will necessarily undermine intrinsically valuable things (e.g., human dignity). My analysis shows that the debate on intrinsic value places serious constraints on claims about the intrinsic badness of HET. More specifically, the analysis shows that bioconservative arguments are, for the most part, inconsistent, misconceived, and overly speculative. Enhancement interventions cannot be bearers of intrinsic value on any of its plausible understandings, and, even if we could grant such a possibility, there are no compelling reasons to presume that the intrinsic value of HET would be necessarily negative. As a result, claims regarding their moral impermissibility are unwarranted.

## Introduction

Some authors in the human enhancement debate hold that human enhancement technologies (HET) are intrinsically bad and hence morally impermissible.^1^ For example, prominent bioconservatives typically claim that we might agree about improvements, safety, the fairness of distribution etc., and still be morally hesitant about the permissibility of HET (Fukuyama 2002; Kass 2003; Sandel 2004). This unease about biotechnologies points to something of ethical significance, pertaining to the essence of the activity itself (Kass 2003). Namely, if objections suggesting the intrinsic badness of HET are plausible, empirical questions (such as risk and safety) would become less relevant in accounting for the moral permissibility of HET. Bioconservatives may take an even stronger stance by adopting the so-called *bioconservative thesis*, which states that if HET are intrinsically bad, such interventions should never be permitted, even if they are safe, reliable, and justly distributed.^2^ The claims about HET's intrinsic badness, together with implications for their moral permissibility, are robust and influential—impacting the overall discussion and perception of these technologies. Therefore, they require a deeper examination.

Although bioconservatives explicitly claim that HET are wrong in a nonconsequentialist sense, they do not explicate this claim on a deeper philosophical level—such as by adhering to a theory of intrinsic value. It seems that their understanding of intrinsic badness is commonsensical, but much is left to speculation. In order to reduce speculation and improve the discussion, we need to apply more rigorous standards. My analysis proceeds in two distinct but interconnected steps. First, I examine the claim that HET are intrinsically bad by looking into philosophical theories of intrinsic value. I bring forth some pertinent understandings of what it means for a thing to have intrinsic value (Moore 1993; Korsgaard 1983), what are the primary senses (Kagan 1998), as well as different valences of intrinsic value (Zimmerman 2001). Second, I investigate how these views map onto typical bioconservative arguments about intrinsic badness and moral permissibility of HET. My analysis shows that the debate on intrinsic value places serious constraints on these claims, i.e., they appear unwarranted.^3^

The article explicitly looks into three variants of bioconservative arguments about the intrinsic badness of HET: (i) HET are bad purely in virtue of the way they are—owing to their unnaturalness; (ii) the pursuit of HET (“the desire for mastery”) indicates an intrinsically bad disposition to act; and (iii) HET will necessarily undermine intrinsically valuable things. I challenge each of these variants by leaning on some of the well-established views in the debate on intrinsic value. In particular, I identify intrinsic and relevant nonintrinsic (relational) properties of HET to determine whether one could (and should?) plausibly ascribe negative intrinsic value to HET. Although some bioconservative concerns are relevant in their own right, I argue they are, for the most part, inconsistent, misconceived, and overly speculative to prove HET are problematic in virtue of their intrinsic properties.

Concerning the first variant, I show that we cannot consistently claim across cases that unnaturalness is necessarily a bad property, and if we are concerned about the *disruption* of the natural, the claim about the intrinsic badness of HET is still not decisive. I then reject the idea that the desire for mastery has intrinsic or relational properties that are able to ground the negative intrinsic value of HET, thus undermining the second variant. Finally, contrary to the third variant, necessary consequences that affect intrinsically valuable things cannot ground the (negative) intrinsic value of HET because ‘necessary’ and ‘intrinsic’ are distinct concepts, and we lack theoretical as well as empirical support that HET will in fact undermine any of the valuable aspects of human life.

My overall conclusion is that enhancement interventions cannot be bearers of intrinsic value on any of the plausible understandings and, even if we could grant such a possibility, there are no compelling reasons to suggest that the intrinsic value of HET would necessarily be negative. In addition, even if HET had negative intrinsic value, this would not necessarily entail that they are morally impermissible, since moral permissibility need not entirely depend upon intrinsic value. In effect, the assumption that HET are intrinsically bad does not seem to warrant the moral impermissibility thesis. If HET are indeed not morally impermissible for intrinsicality reasons, we may have cleared the way for a more fruitful discussion. Perhaps HET’s moral permissibility may further depend on other things, such as their contingent properties.

First, I resolve some conceptual ambiguities and draw upon the debate on intrinsic value to clarify what it means for a thing to be intrinsically bad. I also show how this maps onto the enhancement discussion in the form of a preliminary argument. In the third section, I conduct a three-part analysis of key bioconservative arguments, identifying intrinsic (and some nonintrinsic) properties that may be relevant for establishing the (negative) intrinsic value of HET. Fourth, I reflect on the relationship between intrinsic value and the moral permissibility of HET and offer some suggestions for further research of this subject.

## Intrinsic impermissibility thesis, intrinsic value, and enhancement

We can think about the moral impermissibility of human enhancements in at least two senses—enhancements may be considered impermissible regardless of their effects or because of their effects. This is underpinned by the distinction between two types of value, intrinsic and contingent (extrinsic).^4^ Namely, things are intrinsically bad purely in virtue of the way they are, and they are extrinsically bad in virtue of the way they interact with the world. Thus, if we believe HET are bad in themselves, and therefore morally impermissible regardless of their effects, we endorse what I will call the *intrinsic impermissibility thesis*. If, by contrast, we hold that the moral impermissibility of HET is derived from bad consequences, we endorse what I will call the *contingent impermissibility thesis*.^5^ The intrinsic impermissibility thesis is a stronger claim that corresponds with the aforementioned bioconservative view that HET are morally impermissible even if they turned out to be technologically feasible, legal, and safe.^6^ More precisely, the intrinsic impermissibility thesis states that HET are morally bad, and therefore impermissible, *because* of the specific properties that they have intrinsically or necessarily. My main focus is the examination of HET’s supposed intrinsic badness, which has direct implications for the plausibility of the intrinsic impermissibility thesis.^7^ Before turning to this task, I resolve some terminological ambiguities pertaining to key concepts, and examine how this impacts the discussion on human enhancement.

### Conceptual clarifications

To test the intrinsic impermissibility thesis, we need to examine whether the practice of human enhancement, on its most plausible understanding, can be a bearer of *negative* intrinsic value.^8^ This ought to be preceded by establishing conceptually sound notions of human enhancement and intrinsic value, as well as identifying some of their fundamental features. This also involves identifying what exactly is the subject of this ethical evaluation. In bioethics, human enhancement is typically understood as an action or process, “a deliberate intervention, applying biomedical science, which *aims to improve* an existing capacity that most or all normal human beings typically have, or to create a new capacity, by acting directly on the body or brain” (Buchanan 2011, p. 23; emphasis added). This definition has several closely and causally related parts that could all serve as subjects of our analysis. Conceivably, we could analyze separately the biomedical technologies used to perform the intervention, the intervention itself, and the result of the intervention. But here, I will focus on the *intervention* or *activity* itself, while assuming that the biomedical means/technologies are an indispensable part of the intervention.^9^ To determine whether enhancement as an *act* can be intrinsically bad, we need to take a closer look at what it means for a thing to have intrinsic value.

Two senses of intrinsic value seem relevant for our purposes: the value a thing has *in itself* and the value it has *as an end*. The first sense is the standard, predominant interpretation of intrinsic value as value a thing has ‘for its own sake’, ‘as such’, or ‘in its own right’ (Zimmerman 2001; Ronnow-Ramussen et al. 2005, p. xiii).^10^ Since this kind of value does not depend upon anything else, we say it is nonderivative (as opposed to derivative value that a thing derives from something else). In other words, it is the value a thing has solely in virtue of its intrinsic (nonrelational) properties. In the second sense, intrinsic value need not entirely depend upon the object’s intrinsic properties—it can depend in part upon the object’s nonintrinsic relational properties (Kagan 1998, p. 280).^11^ This occurs when something that is extrinsically good is valued as an end because of the “interest that someone took in it, or the desire that someone had for it, for its own sake” (Korsgaard 1983, p. 172). Take *uniqueness*, a nonintrinsic relational property contributing to the intrinsic value of an object such as a work of art. Similarly, intrinsic value could be ascribed based on *causal* properties. For instance, a car’s capacity to perform at a particular speed can be found valuable in itself without the car ever being driven or there being an intention to drive it.^12^ Although this second view of intrinsic value is not without its difficulties, it has its appeal. I will approach both senses of intrinsic value as relevant for my analysis, but will take that the assessment of the first sense has greater analytical weight because of its prevalence.

In addition to the two senses of intrinsic value, we should note that intrinsic value can have at least two valences, positive and negative (good and bad).^13^ In ordinary language, it is standard to understand ‘value’ and ‘valuable’ as something good or favorable. However, in philosophical parlance, it is not uncommon to distinguish between positive and negative value: “the claim that something has value may be predicated not on the judgment that it is good but, for example, on the judgment that it is bad, that is, that its value is the negative one” (Zimmerman 2001, p. 3). Also, intrinsic good and bad come in degrees of intensity, which makes intrinsic value computable.^14^ But when is it that things have intrinsic value? One traditional method for testing whether a particular thing can be a bearer of intrinsic value is Moore’s *method of isolation.* This test asks whether a thing is such that, if it existed by itself in absolute isolation, we would judge its existence to be good or bad (Moore 1993).^15^ Another method runs in reverse to Moore’s isolation test. Namely, the thing is a bearer of intrinsic value if we can imagine it to have value in any or all circumstances—that it carries its value with it, so to say (Korsgaard 1983, p. 171).^16^ Although there is more to both of these proposals, simplified versions will suffice for our current purposes. I will take it that a thing has intrinsic value if we found it valuable in all circumstances, or if nothing else existed in the world. What, then, are the preliminary implications of the intrinsic value debate for the discussion on the intrinsic value of HET?

### Preliminary argument

Keeping all of the above in mind, it seems that human enhancements cannot be bearers of intrinsic value, and, therefore, cannot be intrinsically bad. If, however, they could have intrinsic value, this value is more likely to be positive than negative. My argument rests on four premises.

First, it seems implausible to say that biomedical interventions, in general, have value ‘in themselves’ or ‘as such’, regardless of anything else. This is because their value seems to depend entirely upon external factors, such as purpose or efficacy. Although HE aims to improve specific capacities, this tells us nothing about the intervention’s intrinsic (or extrinsic) value.

Second, HE interventions are unlikely to have value as ends because they are not desired as ends—they are merely a means to other valuable ends. For example, interventions that aim to improve the immune system or cognition are best understood as a means to some other valuable end, such as health or virtue. Even if we equated enhancement with the end result of the intervention, such as improved memory, hearing or empathy, it is still reasonable to say that we want these goods as means to some other end, like a good life or happiness. More precisely, enhancements are merely tools for acquiring all-purpose goods—things that are *necessarily* good, but should not be conflated with *intrinsically* good things—i.e., their value is always consequentially justified.^17^ Thus, it seems that enhancement interventions cannot be bearers of intrinsic value on either of the aforementioned interpretations. This creates a considerable burden for the bioconservative case.

Third, in order to claim that enhancements are bearers of intrinsic value, we would have to show that they have value in any or all circumstances or in absolute isolation. First, it is not at all obvious that biomedical interventions carry their value with them—in all conceivable circumstances—regardless of their interaction with other things. It is essential to know whether an intervention makes a person better or worse off, in order to judge it good or bad. This suggests the value we ascribe to interventions is *entirely* contingent (not intrinsic).^18^ We can easily imagine scenarios in which an intervention to improve one’s hearing or memory would make a person better off, as well as worse off. Looking at the intervention (or its intrinsic properties) in isolation, without taking into account any external factors, reveals nothing about its value. Unless we can determine the value of the intervention regardless of its consequences, it will not be a likely bearer of intrinsic value.^19^ We draw a similar conclusion when we apply the second rationale. If an improvement in cognition was the only thing in existence (i.e., there are no viable targets of implementation), and we still found it valuable, only then would the value count as intrinsic. But ascribing intrinsic value to such interventions is not intuitively appealing, and even if we were to allow it, we would face the following implications.

Fourth, if HET can be bearers of intrinsic value, it follows (according to the two-valences rationale) that this value can be *positive*, as well as negative. In fact, bioconservative claims about the intrinsic badness of HET set the ground for a counterpoint about their possible intrinsic goodness. Conceptually, at least, we have equally good reasons to believe HET are intrinsically good or intrinsically bad. One might even suggest that we, in fact, have conceptually sounder reasons to believe HET are intrinsically good. Namely, if we were to judge HET a priori, it seems more reasonable to ascribe positive value to interventions that are designed to deliver good things such as improving the quality of life.^20^ In principle, we are compelled to accept at least one of the following: either HET *cannot* be bearers of intrinsic value and are, therefore, intrinsically neither good nor bad; or they *can* be bearers of intrinsic value, in which case they can bear both intrinsic goodness and badness.

The debate on intrinsic value places serious constraints on bioconservative views about the intrinsic badness of HET. To determine whether their arguments can overcome such constraints, we need to examine them in more detail, especially in terms of relevant intrinsic and nonintrinsic properties. In what follows, I will inspect three potential sources of intrinsic value, as found in the enhancement debate. The first two concern exclusively the intrinsic and relational properties that could ground negative intrinsic value, while the third relates to the *necessary* consequences that affect intrinsically valuable things.

## Concerns about the intrinsic badness of HET

Bioconservative arguments relating to the intrinsic badness of HET can be expressed in the following three ways. First, HET are thought to be bad purely in virtue of the way they are—owing to their unnaturalness; or because their unnaturalness will disrupt the natural. Second, the pursuit of HET is thought to necessarily indicate (or generate) an intrinsically bad disposition to act—often referred to as the “desire for mastery”. Third, HET may necessarily have bad consequences that will undermine intrinsically valuable things.^21^ In this section, I challenge each of these variants by taking into account previously established understandings of intrinsic value. I place special emphasis on properties that could conceivably ground the ascription of (negative) intrinsic value to HET. Although some aspects of these bioconservative concerns are relevant in their own right, I argue that they are, for the most part, inconsistent, misconceived, and overly speculative to convincingly establish that HET are intrinsically problematic.

### Concern 1: The unnaturalness of HET

The ‘unnaturalness concern’ comes in a stronger and a weaker version: enhancements are bad owing to their unnaturalness alone, or they will, due to their unnaturalness, disrupt the preservation of the natural. Although these two versions raise fundamentally different concerns (intrinsic and contingent), they are closely and causally related, and can be jointly addressed. The goal here is to examine both claims with respect to the two senses of intrinsic value. In other words, I examine whether HET are bad in virtue of their intrinsic properties (such as unnaturalness), and whether they are bad in virtue of their relational properties (such as the capacity to disrupt the natural).

The stronger version views unnaturalness as an intrinsic property of HET because enhancements are deliberate interventions (they do not occur naturally) brought about by artificial means. The ‘unnaturalness concern’ rests on the assumption that the natural is good, sacred, and should be honored, while the unnatural is bad and should be avoided (Sandel 2004, 2007; Kass 2003).^22^ It follows that enhancements—as far as they are unnatural—are bad in themselves. This approach, however, fails to distinguish between the natural and the good—the natural is not always good (e.g., natural disasters), and the unnatural is not always bad (e.g., art) (see e.g., Kamm 2005; Buchanan 2011). Not only is the strong version of the unnaturalness concern conceptually flawed, but it is also inconsistent with common practice. For instance, we rarely object to the use of artificial means in medicine merely because they are unnatural. Bioconservatives have themselves recognized the inconsistency of objecting to the means of enhancement due to their artificiality: “[since] the use of artificial means is absolutely welcome in the activity of healing, it cannot be their unnaturalness alone that upsets us when they are used to make people ‘better than well’” (Kass 2003, p. 21). Although there is a sense that the naturalness of means matters, as Kass notes, the problem of means “lies not in the fact that the assisting drugs and devices are artifacts, but in the danger of violating or deforming the deep structure of natural human activity” (2003, p. 22). Thus, it seems that unnaturalness alone is not an intrinsic property of HET that can ground negative intrinsic value. This brings us to the second part of the unnaturalness concern and the other sense of intrinsic value.

The weaker version is concerned with HET’s capacity to disrupt the natural; this capacity could be a nonintrinsic relational (most likely causal) property that affects HET’s intrinsic value. As I already acknowledged, bioconservatives are not concerned with unnaturalness simpliciter, but rather with the *preservation of the natural* (status quo). Naturally given processes such as natural procreation, the human life cycle and flourishing are inherently precious and should be preserved (President’s Council on Bioethics 2003, p. 288). Enhancements therefore represent a threat to the natural—they can interfere with or override it.^23^ This view suggests that there are necessary consequences (in this case, negative ones) caused by intrinsic properties of enhancement.^24^ Thus, if an object can have value derived from relational properties such as causal properties (e.g., Kagan 1998), this could affect the value HET have as ends.

However, even if all of these claims are true, they do not decisively determine the intrinsic badness of HET. First, we should not conflate necessity with intrinsicality. Necessary consequences do not show that HET are intrinsically bad. Even though necessary consequences can strongly affect moral judgment, their actual value is always consequentially (derivatively or relationally) justified. For instance, pollution is a necessary feature of air travel, but not its intrinsic property, i.e., it does not make flying intrinsically bad. Even if HET were to have necessarily bad consequences, this would not decisively determine their intrinsic value.^25^ Second, even if enhancement’s capacity to disrupt the natural is indeed a casual property that can impact its intrinsic value, this tells us nothing about the *valence* and the *degree* of that value. If value as an end need not be based on intrinsic properties alone, since the object can have value as an end in virtue of some subset of its properties (Kagan 1998, p. 291), this would equally apply to all sorts of nonintrinsic properties. In order to plausibly claim HET are intrinsically bad, we have to show that intrinsic value based upon relational properties (such as the capacity to disrupt the natural) is not only *negative*, but *so* negative that no amount of positive value could justify the use of HET. Conceptually (as argued in the previous section), we have no particular reason to assume HET’s intrinsic value is negative. Empirically, evidence is not yet available to support either positive or negative intrinsic value in HET.

To summarize, regardless of whether our focus is unnaturalness alone or the disruption of the natural, claims about HET’s intrinsic badness are not justified. It does not necessarily follow that HET have negative intrinsic value in virtue of their intrinsic properties, such as unnaturalness. This is because it is conceptually mistaken to equate the unnatural and the bad, as well as inconsistent with common practice to object to biomedical means based solely upon their artificiality. Even if value as an end can be affected by nonintrinsic properties, we have no particular reason to assume this value would be negative in sum. Perhaps the source of intrinsic badness lies elsewhere, e.g., in the very *desire* to pursue enhancements or disrupt the natural.

### Concern 2: Pursuing HET is an intrinsically bad disposition

On this version of the bioconservative view, pursuing HET indicates bad character, i.e., it reveals the possession of an intrinsically bad disposition to act. For instance, Sandel explains we should not be so concerned about enhancements undermining valuable things such as effort or human agency, but instead about the attitude and dispositions that prompt the drive to enhancement. This concerns the problematic aspiration to “remake nature, including human nature, to serve our purposes and satisfy our desires. The problem is not the drift to mechanism but the drive to mastery” (2004, p. 54). This desire is not only detrimental (or instrumentally bad) to our sense of giftedness and humility, but it also indicates (intrinsically) bad character. However, it is far from clear how we should interpret the drive to mastery: does it motivate enhancement, does it constitute it, or is it perhaps identical with it? Still, we can try running these different possibilities against our two main senses of intrinsic value. In this section, I examine whether HET are bad in virtue of their intrinsic properties, such as (indicating) bad character; and whether they are bad in virtue of their relational properties such as desiring mastery.

First, let us consider whether HET are bad in themselves in virtue of indicating bad character. Pursuing HET indicates bad character, i.e., it reveals the possession of an intrinsically bad disposition to act. Thus, if we take bad character as a property intrinsic to practicing enhancement, we could perhaps claim that this grounds the negative intrinsic value of HET. A similar interpretation comes from Buchanan, who explains the “concern that the pursuit of enhancements, independently of its consequences, itself *indicates* bad character” as the expressivist or nonconsequentialist type of character concern (2011, p. 69). By contrast, consequentialist concerns are “predictions that the pursuit of enhancements will *result* in a worsening of our characters” (Buchanan 2011, p. 69). The expressivist concern can be further understood as the claim that a stable desire to enhance is itself a manifestation of vice or, at least, predominantly the expression of a vice (Buchanan 2011, p. 69). But can the agent’s character plausibly ground the value some activity has in itself?

The suggestion that HET are bad in themselves because they are motivated by bad character is flawed in several respects. It is generally mistaken to think about desires, motivations, and character traits as intrinsic properties because they are typically subjective/relational. These properties must be *intrinsic* to an enhancement intervention in order to ground its intrinsic value. However, they are not constitutive of its description or definition, or in any other way part of its intrinsic nature. An agent’s character traits, motives, and desires might count as relevant *nonintrinsic* properties, but surely they do not determine the value that an enhancement intervention has *in itself*. Enhancement critics may *identify* enhancement with the desire for mastery or assume that they are intertwined/closely related. But even then, they would still have to show why desiring mastery is bad in itself—what is intrinsically wrong about taking control over (human) nature (assuming that taking absolute control is even possible)?^26^ The wrongness of such interventions is not self-evident because the ‘hesitation’ and ‘unease’ we may feel about enhancements are not decisive for determining their intrinsic badness.

The concern about mastering our nature may collapse into the previously discussed concern about the value of the natural. Although it is flawed to assume that natural is always good, as I previously showed, bioconservatives seem to be making an implicit claim that deliberate changes to human nature are illicit or immoral: “[to] successfully claim that a change in a person’s nature is intrinsically immoral, we need a premise that there is an obligation to limit ourselves to the capacities provided by evolution” (Lindsay 2012, p. 19). Hence, if there is even the slightest chance of HET being intrinsically bad, because they presumably indicate a bad desire, bioconservatives need to show why mastering our nature is bad in itself. This is yet to be proven, but such a claim seems difficult to sustain.

On the second account of intrinsic value, even if the indication of bad character cannot ground the value of an enhancement intervention in itself, some nonintrinsic relational properties could contribute to its value as an end. Nonintrinsic relational properties such as subjective experience, a manifestation of excellence, causal properties, etc. can contribute to a thing’s intrinsic value (e.g., Kagan 1998). Desiring enhancement (or mastery—assuming they amount to the same thing) as an end would count as relevant subjective experience. This assumption seems prima facie justified since desires are typically relational and desiring a thing as an end is one of the valid ways to ascribe intrinsic value. However, I argued in the first section that it seems most reasonable we desire enhancement as a means to some other end, such as health, virtue, or beauty—not as an end in itself.^27^ Thus, as far as we desire mastery instrumentally, the bioconservative assumption is wrong, and even if we desired mastery as an end, mastering our nature would not be proven necessarily bad.^28^

Furthermore, it is erroneous to equate enhancement with complete mastery. Even if we take mastery to represent a manifestation of excellence, which counts as a relevant nonintrinsic relational property and could contribute to the value a thing has as an end, it does not follow that enhancement is that thing. Enhancement should not be equated with mastery because mastery stands for improving a skill to the point of perfection, whereas enhancement is typically understood as any (and not necessarily the highest) degree of improvement above the norm. A distinction between greater and complete mastery would allow us to show, at best, that enhancement indicates a desire for greater mastery broadly considered, but not necessarily complete mastery. Bioconservative claims seem most plausible when we talk about complete mastery, less so for less than complete mastery. Most advocates of enhancement (except perhaps radical transhumanists) would say that enhancement does not aim for complete mastery (perfection) at all, but merely an improvement on the current state of affairs.^29^ If so, the entire argument from ‘perfection’ might be missing its target.

One might object that enhancement could lead to mastery (or other intrinsically bad things) on similar grounds as it may lead to the disruption of the natural—in virtue of its *causal* properties. Causal properties of an object are relevant for intrinsic value when the object produces or is a means to another valuable object (Kagan 1998, p. 283).^30^ Thus, if enhancement is a means to (or produces) mastery, and mastery is intrinsically bad, then enhancement may produce negative intrinsic value in virtue of its relational/causal properties. However, as I already argued, not only is enhancement not necessarily a means to complete mastery, but complete mastery is not decisively intrinsically bad. If we are, in turn, discussing only greater levels of mastery, in broader terms, the bioconservative argument applies with even less strength.

Even if we grant that the desire for mastery necessarily motivates enhancement and that mastery is intrinsically bad, which in turn affects the intrinsic value of enhancement (in virtue of its causal properties), it does not follow that the value of enhancement as an end is necessarily negative on balance. We would need to show it to be so overwhelmingly bad that it grounds the negative intrinsic value of HET. Since nonintrinsic properties only *contribute* to intrinsic value, their effect is not decisive. Contributive value is commonly understood as the value of a part in an intrinsically valuable whole (Korsgaard 1983). Thus, the bioconservative assumption that the desire for mastery is so bad that it outweighs all other contributing factors is not obviously true and calls for additional support. I argued earlier why such a claim is not conceptually stronger than the claim about the intrinsic goodness of HET, but I offer additional reasons in the next section.

### Concern 3: HET’s necessary consequences

So far, we have examined the possibility of different intrinsic (unnaturalness; bad character) and nonintrinsic (disrupting the natural; desire for mastery) properties grounding the negative intrinsic value of HET. We saw that the debate on intrinsic value poses various challenges and, absent further argument, offers no reason to assume HET are intrinsically bad. However, there is another variant of the bioconservative argument: HET may vitiate intrinsically valuable things; as far as they do so *necessarily,* a relevant concern arises regarding the intrinsic value of HET.^31^ This argument raises two concerns: (i) do HET *in fact* represent a threat to intrinsically valuable things (do they necessarily generate bad consequences), and (ii) even if they do, can necessarily bad consequences ground intrinsic value? Here, I mainly focus on the second concern, showing that HET are not necessarily a threat to intrinsically valuable things (not all HET will generate bad consequences), and even if they are, this does not determine their intrinsic value.

I mentioned earlier that bioconservatives believe enhancements represent an aspiration to remake human nature and take absolute control over our lives. The negative side of this aspiration, according to Sandel, lies in the possibility of destroying the appreciation for the gifted character of human powers and achievements; in other words, we would be missing the sense of life as a gift (2009, pp. 53–54).^32^ Genetic enhancements will “undermine our humanity by threatening our capacity to act freely, to succeed on our own and to consider ourselves responsible—worthy of praise or blame—for the things we do and for the way we are” (2009, p. 78). Similarly, Fukuyama (2002) fears that biotechnologies threaten to undermine our human essence and dignity, and are likely to create a genetic underclass. His argument about human dignity states that enhancement will undermine the grounds for a nonarbitrary claim to equal respect: “What the demand for equality of recognition implies is that when we strip all of a person's contingent and accidental characteristics away, there remains some essential human quality underneath that is worthy of a certain minimal level of respect–call it Factor X” (2002, p. 149).^33^ What we want to protect from future advances in biotechnology is “the full range of our complex, evolved natures against attempts at self-modification. We do not want to disrupt either the unity or the continuity of human nature, and thereby the human rights that are based on it” (2002, p. 172). The question then is whether HET will necessarily undermine values such as giftedness and human dignity and how this relates to their intrinsic value.

Enhancements are not necessarily incompatible with, nor will they necessarily undermine intrinsically valuable capacities such as giftedness and dignity. Several scholars challenged Sandel’s argument stating not only that the deterministic approach to enhancement is false, but that enhancements might in fact improve some capacities we find intrinsically valuable (e.g., Kamm 2005; Savulescu 2009; Buchanan 2011; Lindsay 2012; Hauskeller 2013).^34^ Even if some extreme versions of enhancement might represent threats to intrinsically valuable capacities (perhaps radical transhumanism), it is not a *necessary* feature of enhancements that they do so. It is more important to focus here on the concerns that considerations of intrinsic value bring about: can necessary consequences ground intrinsic value?

Following the standard interpretation of value that a thing might have in itself, it is conceptually implausible to ground intrinsic value on consequences, even if they are necessary. I already mentioned, while addressing the first concern, that necessity and intrinsicality are two distinct concepts. The former concerns the consequences of an act and, *ipso facto*, its value is always derivative. The latter concerns the source of value, which resides in the thing itself. I previously mentioned the example of pollution being a necessary consequence of air travel, without making air travel intrinsically bad. Translated into enhancement terms, even if undermining giftedness/dignity were a necessary consequence of enhancement (imagine their occurrence is intertwined/closely related), this does not make it an intrinsic feature of enhancement—especially not the sort that can ground intrinsic value. If anything, the necessary property could impact the contingent value, but this does not warrant a conclusion about HET’s necessary intrinsic badness.

Let us look more closely at Fukuyama’s argument about the undermining of human dignity. Fukuyama holds that the genetic lottery is inherently unfair, but also profoundly egalitarian, while replacing it with choice threatens to increase the disparity (2002, p. 157). In other words, the natural lottery is not bad despite its necessarily bad properties, while self-modification such as HET is bad for being brought about deliberately. Fukuyama suggests that unfair but egalitarian circumstances (such as being equally subject to nature) are better than unfair and inegalitarian (such as deliberate self-modification). While this is a relevant concern in its own right, it does little work for determining the negative intrinsic value of HET. If the natural lottery is good merely because it is natural, then this argument collapses into the (un)naturalness concern. If the natural lottery is good because it is not as bad as deliberate change, then the concern is not about HET’s intrinsic but rather its contingent properties.

Alternatively, we may take that necessary consequences are relevant for the value a thing has as an end, assuming they count as nonintrinsic relational properties. It is disputable whether consequences can count as nonintrinsic relational properties because consequences are usually distinct from something’s properties. But even if this were possible (for reasons of necessity or causality), challenges similar to those discussed previously would emerge. We need to show that the value as an end would be, in sum, negative—the badness of HET’s nonintrinsic properties (necessary consequences) would have to outweigh all other considerations. I have already argued that this claim is not particularly convincing from a conceptual standpoint; considering the points I make against the necessary badness of HET in this section, it seems even weaker. At any rate, a more substantive claim is needed if we want to argue that HET are intrinsically bad in virtue of their consequences. Critics of enhancement have not offered plausible arguments on this matter so far, and my analysis suggests this can hardly be expected.

In summary, bioconservative arguments grounded in unnaturalness, the desire for mastery, and necessary consequences do not warrant any conclusions about HET’s intrinsic value. They are, as they currently stand, highly speculative, incoherent, and empirically unfounded. Now that we have drawn tentative conclusions about the intrinsic status of HET, we need to explore how this reflects upon the intrinsic impermissibility thesis, presented in the first section.

## The gap between intrinsic badness and moral permissibility?

The examination of HET’s intrinsic status was primarily motivated by its relation to the intrinsic impermissibility thesis, i.e., the claim that HET are morally impermissible regardless of their effects. What do our tentative conclusions about the intrinsic status of enhancement say about its moral permissibility? My analysis showed that considerations of intrinsic value place significant constraints on the intrinsic impermissibility thesis. Ascribing negative intrinsic value to HET, absent further argument, seems unwarranted. Let us, nevertheless, assume that more cogent arguments for HET’s intrinsic badness become available. Would this resolve that they are morally impermissible? Probably not, and here is why.

The intrinsic impermissibility thesis expresses a very general bioconservative stance towards enhancements, but it also assumes a strong connection between a thing’s intrinsic value and its moral permissibility. This assumption, however, is not entirely justified. A thing’s intrinsic value may carry significant normative weight (it can affect moral judgments and the moral status of an object), but, strictly speaking, it does not necessarily determine that thing’s moral permissibility. Things can still be morally bad and permissible (e.g., war). Moral theories organize and relate accounts of the right and the good differently: e.g., duty-based theories place lesser weight on value concepts, while theories of value explain right and wrong action in terms of how they bear on intrinsic value (Timmons 2012, pp. 10–11). Therefore, goodness and badness do not necessarily affect the (im)permissibility of an act.

Consider once more the bioconservative concern about HET being intrinsically bad because they indicate bad character (the desire for mastery). One could argue that the agent’s character and motivation can be relevant for the moral status of an act, but does not affect its moral permissibility. Some scholars have put forward arguments along these lines. For example, Kamm (following Scanlon and Thomson) suggests that the permissibility of an act can be seen as independent from our intentions or dispositions to carry the act out—intentions and attitudes of an agent reflect on the agent’s character but not on the permissibility of their act (Kamm 2005, p. 7). For example, a scientist motivated by the desire for mastery in her quest to find a cure for some nasty disease may not be a good person on every moral account, but we would not find her conduct impermissible. Similarly, Buchanan states that motivation is not the only relevant factor for determining permissibility, given that even bad motivation can result in the right act: “even if it were true that the pursuit of enhancement is always driven solely by bad character, it would not follow from this that enhancement is morally impermissible” because “one can perform the right act as a result of bad motivation” (2011, p. 72). Hence, even if there were reasons to think HET are intrinsically bad, this would not necessarily imply their moral impermissibility.

This is not to say HET are generally permissible. Even if the intrinsic impermissibility thesis is false, and we cannot plausibly claim HET are morally impermissible for intrinsicality-based reasons, they can be impermissible for other reasons. Their permissibility may depend upon potentially bad consequences, i.e., the plausibility of what I have previously called the *contingent impermissibility thesis*. The contingent impermissibility thesis states that HET are morally impermissible insofar as they are reasonably expected to be contingently bad. Unlike the intrinsic impermissibility thesis, the contingent impermissibility thesis is not an absolute claim. It allows for the conditional assessment of HET: *If* HET are bad in their interactions with other things, then they should be deemed morally impermissible. This implies that HET could be morally *permissible* if we could reasonably expect them not to have bad effects. If we endorse this line of reasoning, future research should consider contingent instead of intrinsic factors.^35^ My examination has hopefully cleared the way for a more fruitful discussion focused on the evaluation of the contingent rather than the intrinsic value of HET.

## Conclusion

In this article, my primary aim was to show that notions of intrinsic value place serious constraints on claims about the intrinsic badness of HET. I argued that enhancement interventions are not typical bearers of intrinsic value on any of its plausible understandings. Even if we granted such a possibility, I argued that there were no compelling reasons to accept that the intrinsic value of HET was negative. Additionally, even if HET had negative intrinsic value, this would not warrant an unfavorable verdict about their moral permissibility. The examination of HET’s intrinsic and relevant nonintrinsic properties, such as unnaturalness, the agent’s desire for mastery, and the necessary production of bad consequences, was not decisive in establishing their intrinsic badness. Under scrutiny, most of these concerns collapse into one another, and ultimately into questions about contingent factors that could, in fact, play a prominent role in accounting for the moral permissibility of HET.

## Footnotes

1 Although bioconservatives claim that human enhancement *technologies* are intrinsically bad, it seems odd to think of technologies like medical machines or pills as bad in themselves. Hence, I will assume that bioconservatives believe human enhancement as an *action*, brought about by biotechnological means, is intrinsically bad. Henceforth, I will use the terms HET and human enhancement as roughly amounting to the described intervention.

2 Some scholars may think that this formulation of the bioconservative thesis does not represent bioconservative views accurately. Milder interpretations of their views include “a general plea for caution” or simply a suggestion that “implementing HET is unwise”, but with no repercussions for its moral permissibility (e.g., Hauskeller 2013). I, however, believe that bioconservative views are too robust and influential to be taken lightly.

3 My analysis is limited to the evaluation of intrinsic badness of HET from the perspective of philosophical theories of intrinsic value and the evaluation of internal consistency of bioconservative arguments. As the anonymous reviewer for this journal rightly noted, there are other approaches that could be taken here. Many terms used in the intrinsic value discussion (e.g., nature, dignity, intrinsic badness, etc.) are borrowed from the rich tradition of natural law reasoning or Aristotelian metaphysics school (see, e.g., Murphy 2019). Although natural law theory may offer valuable insights on this subject, my mode of argument follows the cited authors and is analytical philosophy. For a discussion on biotechnologies and natural law see, e.g., Anderson and Tollefsen (2008); for some theological aspects see, e.g., Boer and Fischer (2013).

4 The traditional distinction between intrinsic and extrinsic value maps onto my concerns about intrinsic and contingent reasons for or against HE. I prefer using the term ‘contingent’ because I do not think any knock-on consequences or implications are necessary except for those that follow from intrinsic properties.

5 By ‘consequences’ I understand a broad range of effects that are necessarily or contingently brought about by enhancement. They include affecting societal values like justice, rights, and virtue, practical aspects like distribution and regulation, as well as welfare/states of affairs. I will touch upon the relevance of the contingent impermissibility thesis in the last section.

6 Here, I follow Douglas’ reading of the bioconservative thesis: “[e]ven if it were technically possible and legally permissible for people to engage in biomedical enhancement, it would not be morally permissible for them to do so” (2008, p. 228).

7 One could also endorse a weaker version of the intrinsic impermissibility thesis stating that intrinsic badness of a practice p provides a strong reason against p’s permissibility. Even if this is the case, my conclusions apply.

8 Negative intrinsic value has the same meaning as intrinsic disvalue or intrinsic badness.

9 As I mentioned, it does not seem reasonable to think of biomedical means/technologies like medical machines or pills as bad in themselves. Also, equating HE with the end result of the process imposes the risk of normative ladenness. Namely, this entails that enhancement makes us better off by definition—if an intervention does not make us better off, it cannot be considered enhancement. As far as our goal is the evaluation of the intervention as such, we should refrain from equating human enhancement with its end result.

10 Some scholars warn that to ascribe intrinsic goodness to something is not to say that it is valued for its own sake, but that it has goodness in itself—it refers to the source of goodness rather than the way we value it in (Korsgaard 1983).

11 As Kagan explains, “if something does have value as an end, then there is reason to ‘promote’ it, to try to produce the valuable object, or perhaps to preserve and maintain it; we sometimes say that the world is better off ‘as such’ for the existence of the valuable object” (1998, p. 279). We can assume that the opposite applies to objects with negative intrinsic value: we should not try to produce, preserve and maintain them, and the world seems worse off ‘as such’ for their existence.

12 Also, certain skills that are instrumentally valuable could be valued in themselves—an ability such as superb cooking could be valued even if one never gets to exercise it—but its intrinsic value depends in part upon its usefulness. For a list of such relational properties and detailed examples, see Kagan (1998).

13 Some things have no value—they are neither good nor bad, positive nor negative (Timmons 2012, p. 8).

14 For a detailed discussion on degrees of intrinsic value and its computability, see, for example, Zimmerman (2001) Chapter 5.

15 For a detailed analysis of Moore’s isolation test, see, for example, Zimmerman (2001), Chapter 5.

16 By contrast, if a thing is not good in any and all circumstances, its goodness is extrinsic—derived from or dependent upon circumstances (Korsgaard 1983, p. 171).

17 All-purpose goods such as memory, intelligence, patience, empathy, and morality, “are traits that are valuable regardless of which kind of life a person chooses to live—valuable on all plausible conceptions of well-being” (Savulescu et al. 2011, p. 11). Also, things that have *necessary* value (such as all-purpose goods) may be a relevant target of this discussion, but I engage with this later.

18 This point is in line with the instrumentalist approach—a predominant view in philosophy of technology which sees technologies as neutral means for achieving human goals (as opposed to substantivism which gives technologies a deterministic role in society) (Borgmann 1984).

19 Thus, unless the improvement of cognition is valuable despite scenarios where it makes a person worse off, this action is not intrinsically valuable. Conversely, we would have to show that intrinsic disvalue is present even when an intervention is perfectly safe and makes someone better off in a relevant way.

20 Perhaps the most reasonable thing to assume from a conceptual point of view is that HET are neither good nor bad, but neutral.

21 This categorization approximately corresponds to the object, intention, and circumstances relevant for evaluating morality of a human act. In its strict sense, intrinsic badness is only applicable to the object (such as the one in the first concern). However, the other two variants aim to identify *potential* objects of intrinsic value within bioconservative concerns and inspect how that corresponds with standard interpretations of intrinsic value.

22 The concept of human nature used here equates nature with physicality or biology. It is sometimes possible to distinguish between physical and metaphysical aspects of human nature. However, it is not in the scope of my paper to engage with those views.

23 The idea of preserving the natural and human nature is paradoxical because these things are constantly changing and enhancements can become essential for preserving the status quo (Harris 2007; Buchanan 2011). Also, it is erroneous to think that most enhancements would change human nature since this would require a modification of a central trait on a population level (Daniels 2009). I will therefore focus on challenges prompted by the debate on intrinsic value.

24 I will address specific aspects of necessary consequences caused by intrinsic properties of HET, as well as their relational properties, later in the article.

25 This issue will be addressed in more detail under the third concern.

26 Actual mastery and the desire for mastery are two distinct concepts (Kamm 2005). It is improbable that actual mastery (absolute control over nature, including human nature) is attainable because while most enhancements involve changes in an individual’s nature, they do not have the potential to overthrow human nature entirely (Daniels 2009).

27 Some scholars also suggested we could master nature as a side effect, without desiring it as an end (Kamm 2005, p. 6).

28 I address this further later in the paper.

29 If the exact target is radical transhumanism, then the argument is inapplicable to *all* enhancements, since most entail minor to moderate changes.

30 Kagan’s example of such a causal property is the capacity of a racing car to perform at a particular speed. We might think the car is intrinsically valuable in virtue of its relational properties, including its causal properties (1998, p. 284).

31 It is important to distinguish between necessarily having/generating bad consequences and contingently generating consequences that are themselves necessarily bad. What I mean here is the former.

32 Other examples of intrinsically valuable things threatened by enhancements include humility, spontaneity, the mystery of life, openness to the unbidden, human flourishing, etc. Hauskeller explains this concern in greater detail by claiming that attempts to make better people seem to be detrimental to humility, a virtue that Sandel highly values. Hence, the main concern is the harm we might inflict on ourselves as enhancers, by allowing the reign of our inherent drive to mastery (Hauskeller 2013, p. 167). Harm, however, is not a question of intrinsic value.

33 Factor X is a combination of qualities such as “the possession of moral choice, or reason, or language, or sociability, or sentience, or emotions, or consciousness, or any other quality that has been put forth as a ground for human dignity” coming together in a whole (Fukuyama 2002, p. 171).

34 For example, Kamm (2005) argued that valuable things promoted by enhancements (e.g., health, virtue, beauty) are not incompatible with other things valued in themselves (e.g., spontaneity, giftedness, human flourishing). The deterministic effect of enhancements was challenged by Savulescu (2009, 2010), Buchanan (2011), and Lindsay (2012). Some weak points of the argument from giftedness were analyzed by Hauskeller (2013).

35 A similar idea comes from Kamm, as she suggests that in accounting for the permissibility of an act: “[w]e will have to decide whether particular changes are permissible independently of the aims, attitudes, and dispositions of agents who act” (2005, p. 7).
